# *In vitro* and *in vivo* anti-tumor effects of brexpiprazole, a newly-developed serotonin-dopamine activity modulator with an improved safety profile

**DOI:** 10.18632/oncotarget.26949

**Published:** 2019-05-28

**Authors:** Shuhei Suzuki, Masahiro Yamamoto, Keita Togashi, Tomomi Sanomachi, Asuka Sugai, Shizuka Seino, Takashi Yoshioka, Chifumi Kitanaka, Masashi Okada

**Affiliations:** ^1^Department of Molecular Cancer Science, Yamagata University School of Medicine, Yamagata 990-9585, Japan; ^2^Department of Clinical Oncology, Yamagata University School of Medicine, Yamagata 990-9585, Japan; ^3^Department of Ophthalmology, Yamagata University School of Medicine, Yamagata 990-9585, Japan; ^4^Research Institute for Promotion of Medical Sciences, Yamagata University Faculty of Medicine, Yamagata 990-9585, Japan

**Keywords:** brexpiprazole, serotonin-dopamine activity modulator, drug repositioning, drug repurposing, survivin

## Abstract

From the perspective of psycho-oncology, antipsychotics are widely used for patients with cancer. Although some antipsychotic drugs have anti-tumor effects, these antipsychotic drugs are not applicable for cancer patients because of their severe side effects. Brexpiprazole, a novel serotonin-dopamine modulator with an improved side effect profile, was developed as a drug that is structurally and pharmacologically related to aripiprazole, which was reported to have anti-cancer effects. However, it remains unknown whether brexpiprazole has anti-cancer effects. In this study, we examined whether brexpiprazole has anti-tumor effects in cancer cells and cancer stem cells (CSCs) of glioblastoma, pancreatic cancer, and lung cancer. Brexpiprazole suppressed cell growth and induced cell death in the cancer cells and the CSCs, and decreased the CSC properties of the CSCs. Brexpiprazole did not exert any cytotoxic effects on non-cancer cells at the anti-cancer effect-inducing concentration. In the cancer cells and the CSCs, brexpiprazole reduced the expression of survivin, an anti-apoptotic protein, whose reduction sensitizes tumor cells to chemotherapeutic reagents. In the preclinical model in which pancreatic CSCs were subcutaneously implanted into nude mice, brexpiprazole suppressed tumor growth, in addition to reducing the expression of Sox2, a marker for CSCs, and survivin. This suggests that brexpiprazole is a promising antipsychotic drug with anti-tumor effects and an improved safety profile.

## INTRODUCTION

Cancer is one of the leading causes of death [1]. As cancer patients are forced to endure poor clinical courses, at least 25–30% of patients with cancer have psychological problems, including depression, anxiety, stress-related syndromes, adjustment disorders, sleep disorders, and delirium [2]. Moreover, cancer patients also develop psychological and neurological problems due to chemotherapy against cancer [3]. Therefore, in order to ameliorate these symptoms, psychological drugs are widely given to patients with cancer [2, 4–6]. For example, aripiprazole, a dopamine partial agonist, is used for the management of delirium and emesis in cancer patients [7–9], and olanzapine, a multi-acting receptor-targeting antipsychotic drug, is given as an anti-emetic drug for chemotherapy-induced nausea and vomiting [10–12].

From the point of view of drug repositioning or repurposing, several psychological drugs have been reported to have anti-cancer effects such as the anti-dopaminergic drug thioridazine, and the tricyclic antidepressants (TCAs) imipramine and chlorimipramine [13–16]. Although thioridazine has promising therapeutic potential [13, 17–20], it is no longer available for clinical use because of its severe cardiac side effects such as *torsades de points* [21–23]. TCAs have largely been replaced by selective serotonin reuptake inhibitors, which have a favorable side effect profile. We previously reported the anti-cancer effects of aripiprazole and olanzapine, which are used for cancer patients with fewer side effects [24, 25]. However, olanzapine sometimes causes intolerable sedation [12] and aripiprazole causes akathisia [26, 27]; therefore, antipsychotic drugs that have anti-cancer effects with an improved side effect profile are required.

Brexpiprazole is a new antipsychotic agent for depression and schizophrenia. Brexpiprazole was developed as a drug that is chemically and pharmacologically related to aripiprazole. Brexpiprazole is a partial agonist of serotonin receptor 1A (5-HT_1A_) and dopamine receptor D2 (D2), and an antagonist of serotonin receptor 2A (5-HT_2A_) and noradrenaline alpha_1B/2C_ receptors, thus it acts as a serotonin-dopamine activity modulator, similar to aripiprazole [28]. Although brexpiprazole shares its pharmacological activity with aripiprazole, it has a better side effect profile than aripiprazole due to its lower intrinsic activity at the D2 and dopamine receptor D3 [26, 27, 29]. Brexpiprazole is expected to have anti-cancer activity because of its similarity to aripiprazole, but it remains unknown how its pharmacological difference from aripiprazole affects its anti-cancer activity. In this study, we examined the effects of brexpiprazole on cancer cells and cancer stem cells (CSCs) of glioblastoma, lung cancer, and pancreatic cancer, as well as its toxicity in non-cancer cells.

## RESULTS

### Brexpiprazole inhibits growth and is cytotoxic to cancer cells, including CSCs, but not to normal cells

**Figure 1:**
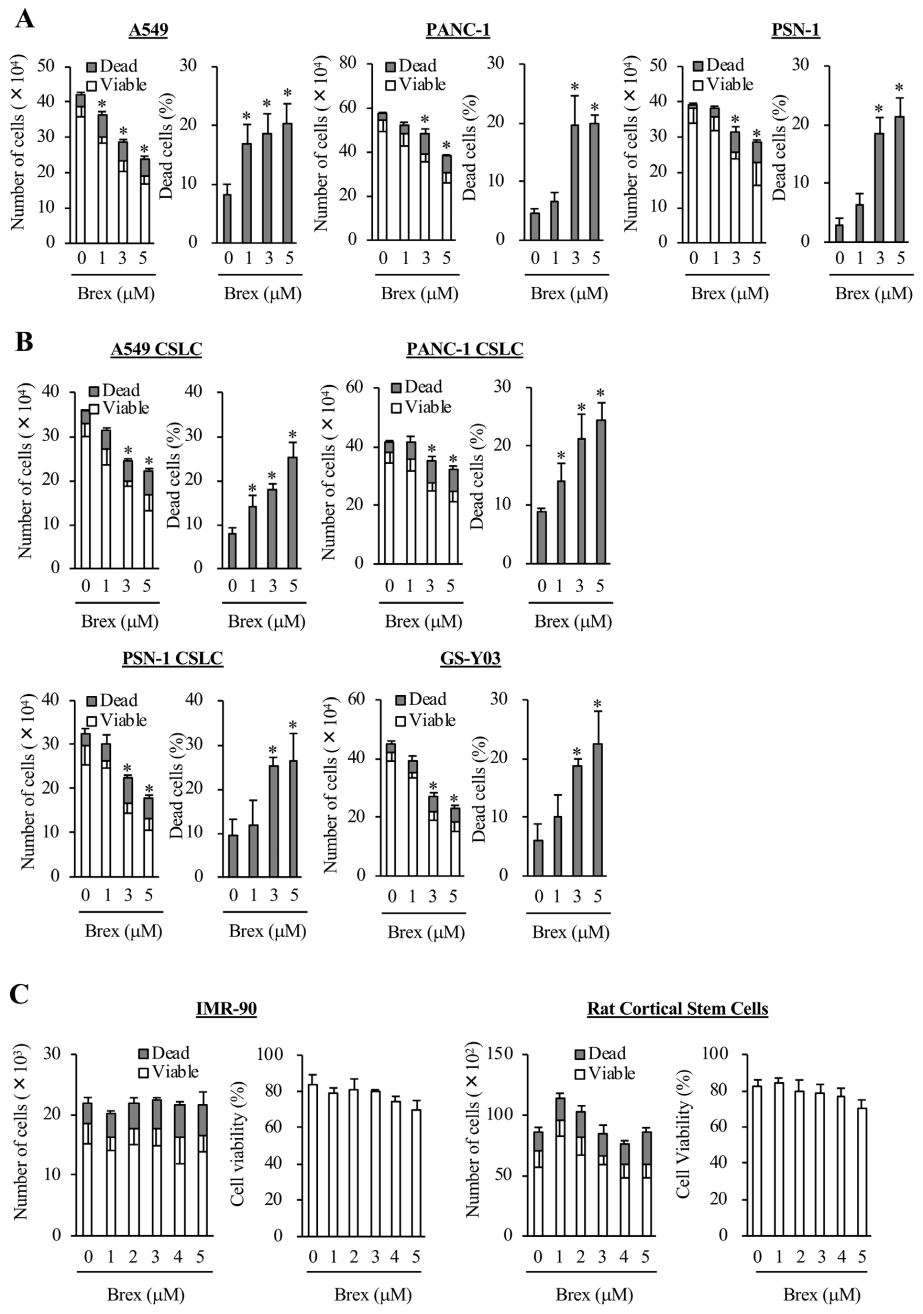
Brexpiprazole suppresses the growth of cancer cells and cancer stem cells, and induces cell death without marked toxicity to normal cells. Cancer cells (**A**), CSCs (**B**), and non-cancer cells (**C**) were treated with brexpiprazole (Brex) at the indicated concentrations for 3 days, and were then subjected to cell viability assays. The numbers of total (viable and dead) (left panels) and percentage of dead cells (right panels) are shown. The number of seeded cells was 1 × 10^5^ cells in (A) and (B), 1 × 10^4^ cells in the left panels of (C), and 5 × 10^3^ cells in the right panels of (C). Values represent the mean ± SD from triplicate samples of a representative experiment repeated three times with similar results. ^*^p < 0.05.

To examine whether brexpiprazole has inhibitory effects on cancer cell lines, three representative cancer cell lines (A549, PANC-1, and PSN-1) were treated with brexpiprazole for 3 days, and then subjected to cell viability assays. Brexpiprazole induced cell death and growth inhibition in the three cell lines (Figure 1A). Next, we examined whether brexpiprazole has inhibitory effects on CSCs. We treated four representative CSCs (A549 CSLC, PANC-1 CSLC, PSN-1 CSLC, and GS-Y03) with brexpiprazole, and they were subjected to cell viability assays. Brexpiprazole induced cell death and growth inhibition in the CSCs (Figure 1B). We also examined the toxicity of brexpiprazole in non-cancer cells. We treated non-cancer cells (normal human fibroblasts [IMR-90] and rat cortical stem cells) with brexpiprazole for 3 days, and then subjected them to cell viability assays. Brexpiprazole was not toxic to the normal cells at the examined concentrations (Figure 1C). These results suggest that brexpiprazole is not toxic to normal cells, but has cancer cell- and CSC-specific cytotoxic and growth-inhibitory effects.

### Brexpiprazole decreases CSC properties

**Figure 2:**
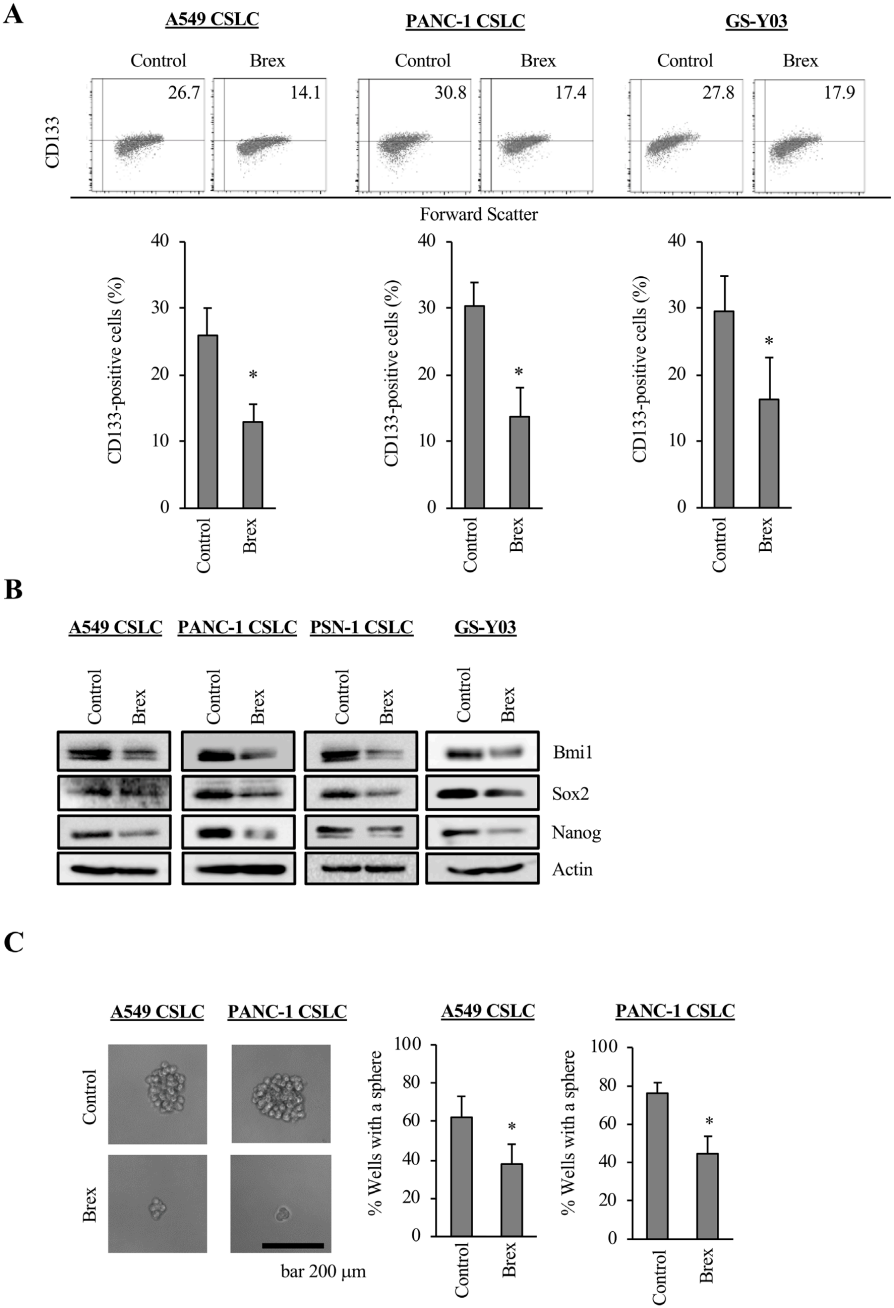
CSCs lose their CSC properties after brexpiprazole treatment. CSCs treated or not treated with 3 μM brexpiprazole (Brex) for 3 days were subjected to flow cytometric analysis for CD133 expression (**A**), immunoblot of indicated proteins (**B**), and the sphere formation assay (**C**). In (A) and (C), the data represent means + SD from 3 independent experiments. ^*^p < 0.05. In (A), representative flow cytometric plots together with the percentage of CD133-positive cells are shown. In (C), the scale bar indicates 200 μm.

As some antipsychotic drugs were reported to decrease the stemness of CSCs [13, 24, 25], we examined whether brexpiprazole similarly decreases the CSC properties of CSCs. We treated CSCs with brexpiprazole for 3 days, and then subjected them to flow cytometric analysis to evaluate the decrease in cell surface CD133, a marker of stem cells. Brexpiprazole reduced the proportion of CD133-positive cells (Figure 2A). We next examined whether brexpiprazole reduces the expression of stem cell markers, such as Bmi1, Sox2, and Nanog, in the CSCs. Brexpiprazole reduced the expression of the stem cell markers (Figure 2B). We then investigated whether brexpiprazole affects sphere formation to evaluate self-renewal ability, which is one of the properties of CSCs. Brexpiprazole reduced the sphere formation ability of CSCs (Figure 2C).

### Brexpiprazole decreases survivin expression and sensitizes CSCs to chemotherapeutic drugs

**Figure 3:**
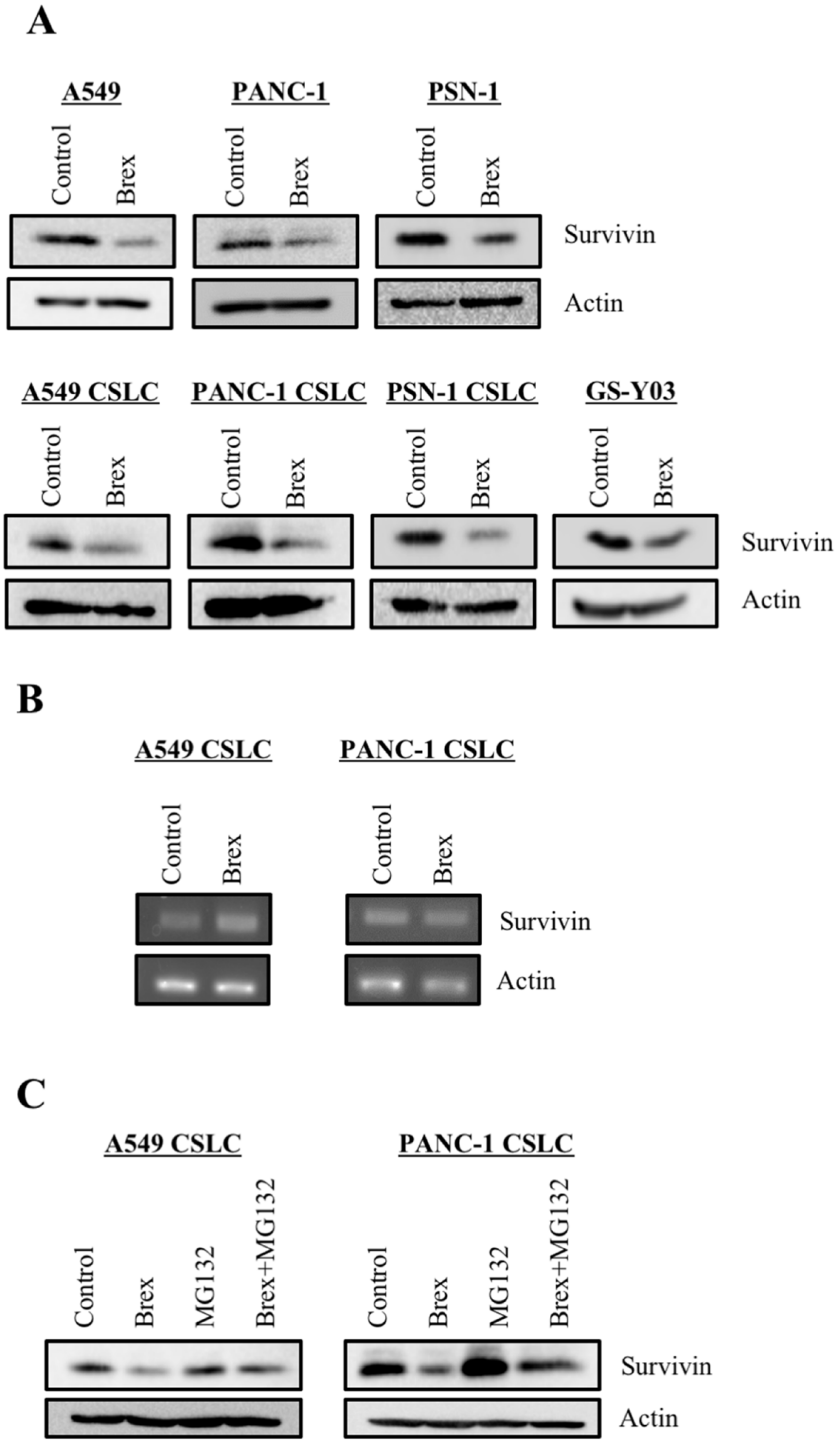
Brexpiprazole reduces the expression of survivin. The cells treated or not treated with 3 μM brexpiprazole (Brex) for 3 days were subjected to immunoblot analysis of the indicated proteins (**A**) and to reverse transcription-PCR analysis of indicated mRNAs (**B**). The cells treated or not treated with 3 μM brexpiprazole for 3 days and subsequently treated or not treated with 10 μM MG132 for 8 hours were subjected to immunoblot analysis of the indicated proteins (**C**).

**Figure 4:**
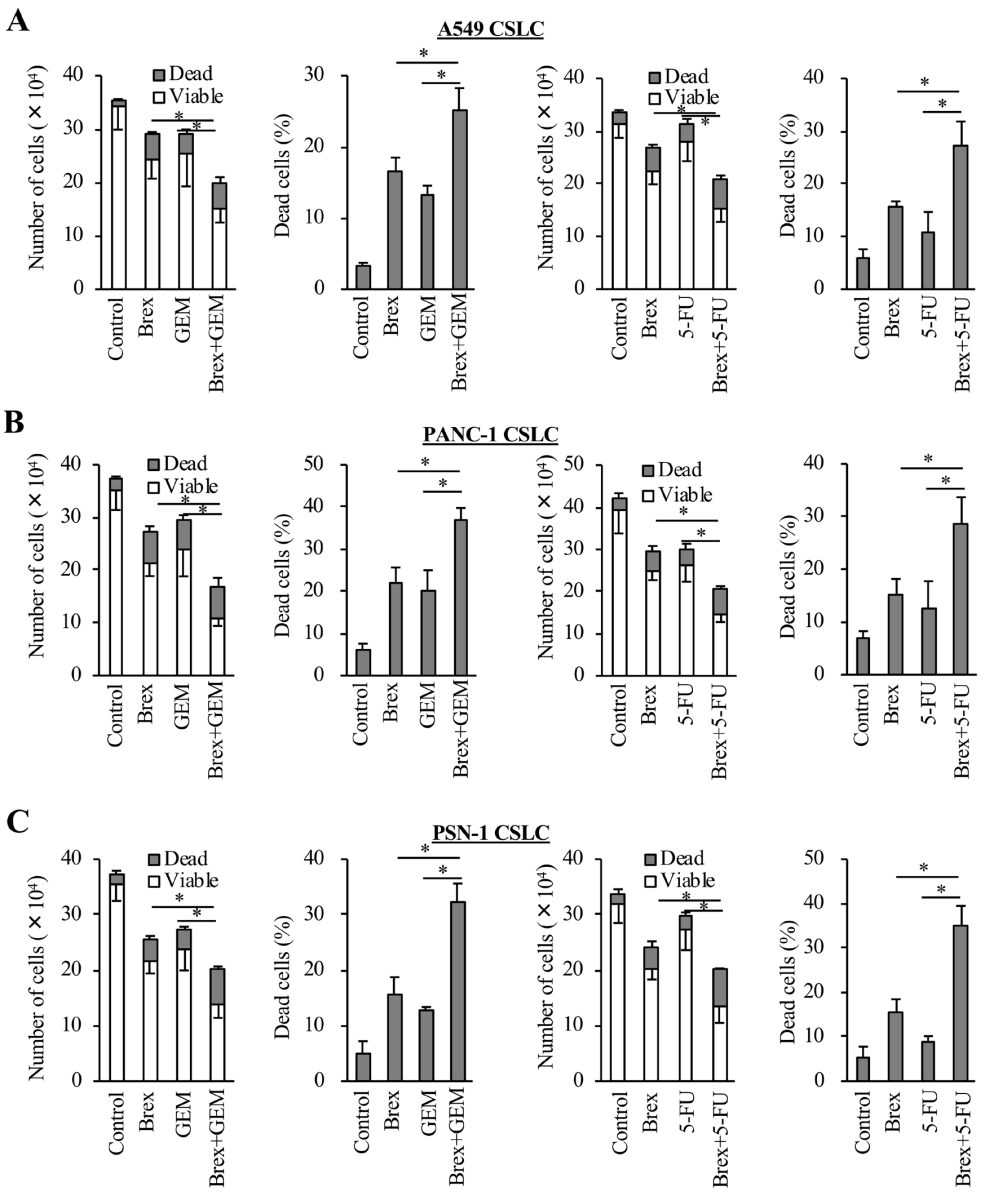
Brexpiprazole sensitizes CSCs to cytotoxic chemotherapeutic agents. The CSCs seeded at 1 × 10^5^ cells per well were treated or not treated with gemcitabine (GEM) or 5-FU in the presence or absence of brexpiprazole (Brex) for 3 days, and then subjected to cell viability assays for the numbers of viable and dead cells (first and third panels from the left), and the percentage of dead cells (second and fourth panels from the left) (**A**–**C**). In (A) – (C), the concentration of brexpiprazole was 3 μM for all cells, the concentration of gemcitabine was 0.25 μM for A549 CSLC cells, 0.5 μM for PANC-1 CSLC cells, and 0.2 μM for PSN-1 CSLC cells, and the concentration of 5-FU was 0.5 μM for A549 CSLC cells, 5 μM for PANC-1 CSLC cells, and 1 μM for PSN-1 CSLC cells. In (A) – (C), values represent the mean ± SD from triplicate samples of a representative experiment repeated three times with similar results. ^*^p < 0.05.

As we previously demonstrated that antipsychotics, such as olanzapine and aripiprazole, reduce the expression of survivin, which is highly expressed in CSCs and has anti-apoptosis effects [24, 25], we examined the expression of survivin in the cancer cells treated with brexpiprazole. Brexpiprazole reduced the expression of survivin in all cell lines. (Figure 3A). The mechanism of survivin reduction by antipsychotics is unknown [24, 25]; therefore, we next explored the regulation of survivin expression by brexpiprazole. Brexpiprazole did not decrease survivin mRNA expression (Figure 3B), suggesting the involvement of a post-translational mechanism. To investigate if brexpiprazole affects the degradation of survivin, we treated the cells with MG132, a proteasome inhibitor, in the presence or absence of brexpiprazole. MG132 treatment partially rescued the reduction of survivin by brexpiprazole (Figure 3C), suggesting that degradation by proteasomes plays a role in the reduction of survivin induced by brexpiprazole. As the reduction of survivin reportedly sensitizes CSCs to chemotherapeutic reagents [24, 25, 30, 31], we examined whether brexpiprazole also sensitizes CSCs to chemotherapeutic reagents. CSCs were treated or not treated with chemotherapeutic agents in the presence or absence of brexpiprazole, and then subjected to cell viability analysis. Brexpiprazole sensitized CSCs to 5-FU and gemcitabine (Figure 4).

### Anti-tumor effects of brexpiprazole *in vivo*

**Figure 5:**
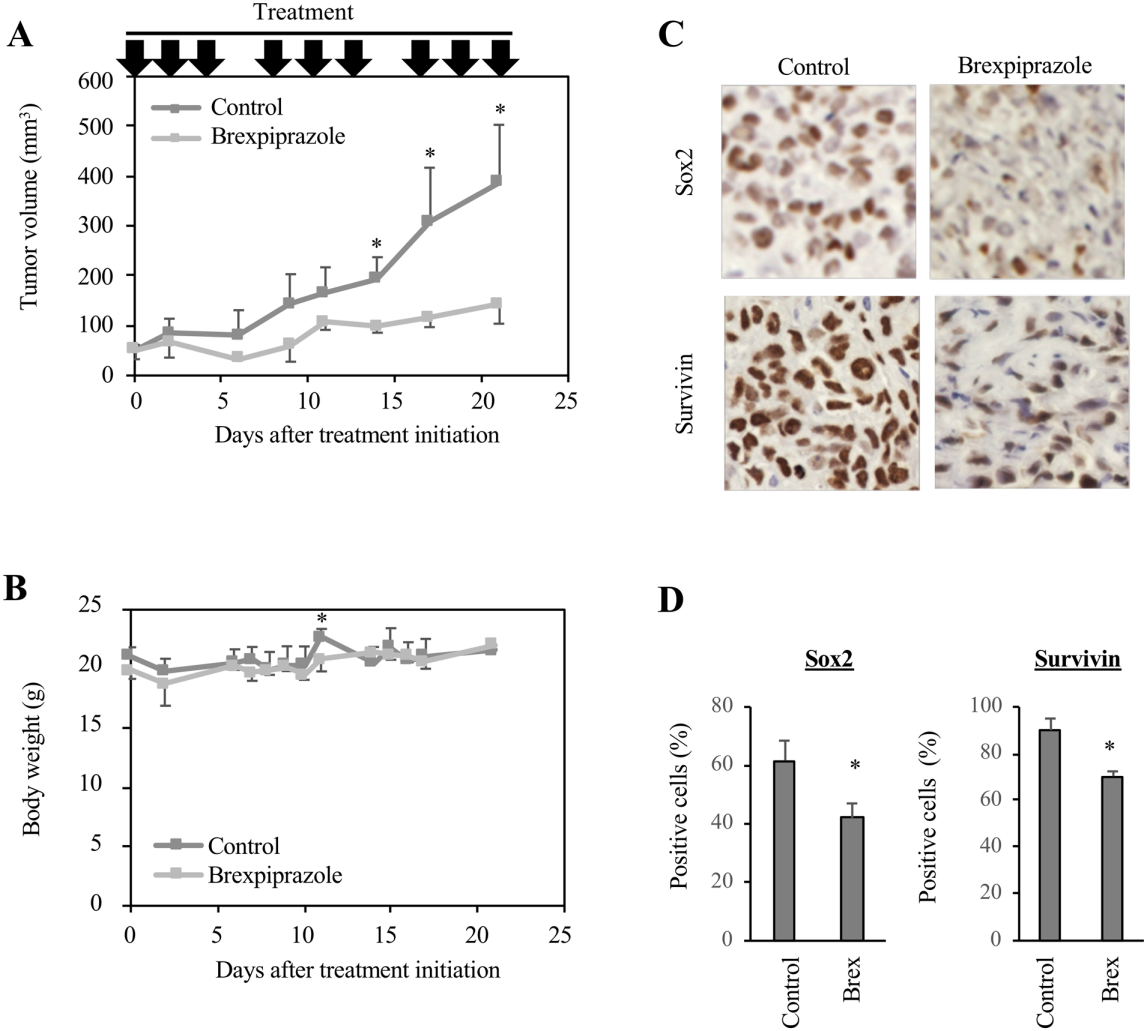
Brexpiprazole suppresses tumor growth *in vivo* and reduces the expression of Sox2 and survivin. PANC-1 CSLC cells were subcutaneously implanted into mice. After confirming tumor formation, drug administration was initiated 10 days after implantation, and the mice were treated or not treated with brexpiprazole (0.1 mg/kg body weight, intraperitoneal injection, three times a week). The tumor volume (**A**) and mouse body weight (**B**) were measured, and the results are shown in the graphs as means ± SD. ^*^p < 0.05. Immunohistochemistry for Sox2 and survivin (**C**), and quantification of the percentage of Sox2- and survivin-positive cells (**D**). In (D), three representative images were taken for each tumor nodule, and the percentage of Sox2- or survivin-positive cells was calculated. The graphs show means + SD. ^*^p < 0.05.

Lastly, to explore the clinical applicability of brexpiprazole to cancer treatment, we evaluated the anti-tumor effects of brexpiprazole *in vivo*. Mice in which PANC-1 CSLC cells were subcutaneously transplanted were treated with brexpiprazole. Brexpiprazole significantly suppressed the growth of the tumors (Figure 5A), but did not alter the body weight (Figure 5B). No notable adverse effects were observed. The expression of Sox2, a representative stemness marker, and survivin was reduced by brexpiprazole administration (Figure 5C and 5D).

## DISCUSSION

Several studies have demonstrated that antipsychotic agents have anti-cancer potential [13–16]. We previously reported that aripiprazole, a serotonin-dopamine activity modulator, has anti-cancer activity [24]. Brexpiprazole is a newly-developed serotonin-dopamine activity modulator that is structurally and pharmacologically related to aripiprazole with a better side effect profile because of its lower intrinsic activity at dopaminergic receptors [26, 27, 29]. However, it remains unknown whether brexpiprazole has anti-cancer activity. This study revealed that brexpiprazole has anti-tumor effects by reducing the properties of CSCs and expression of survivin, an anti-apoptotic protein.

In the previous study, the anti-cancer effects of aripiprazole were explored only *in vitro* [24]; therefore, the anti-tumor effects of serotonin-dopamine activity modulators on CSCs in preclinical mouse models were unknown. In this study, brexpiprazole significantly suppressed the growth of the subcutaneously implanted pancreatic CSCs. Furthermore, it reduced the expression of Sox2, a marker of CSCs, and survivin, an anti-apoptotic protein. This suggests that brexpiprazole can suppress the CSC properties of the tumors *in vivo*. Suppression of survivin sensitizes tumor cells to chemotherapeutic reagents [25, 30, 31], and in this study, brexpiprazole sensitized the CSCs to chemotherapeutic reagents *in vitro*. Therefore, brexpiprazole is expected to act as a chemosensitizer. These data from the preclinical mouse model can facilitate the clinical application of brexpiprazole to patients with cancer.

Brexpiprazole shares its spectrum of targeted receptors with aripiprazole, but its affinity and effects on these receptors differ from those of aripiprazole, leading to the different profile of adverse and therapeutic effects [27, 32]. In this study, brexpiprazole at the concentration effective for cancer cells and CSCs did not suppress cell growth or induce cell death in normal human lung fibroblasts or rat cortical stem cells. Moreover, brexpiprazole at the dosage that significantly suppressed the growth of subcutaneously implanted CSCs did not alter the body weight or cause other notable side effects. Taking into account dose conversion from mice to humans [33], the dose we used in the experiment (0.1 mg/kg) corresponds to 0.00813 mg/kg as a human equivalent dosage. Although the dosage was lower than that clinically used for humans (0.0167 – 0.0667 mg/kg), brexpiprazole was effective in mice. Of note, brexpiprazole was reported to cause fewer adverse effects than aripiprazole; akathisia and insomnia occur 50% less frequently than with aripiprazole [27, 32, 33]. Thus, due to its improved safety profile, brexpiprazole is a promising candidate antipsychotic drug with anti-tumor effects.

Although we previously reported that antipsychotic drugs reduce the expression of survivin in cancer cells [24, 25], the detailed mechanism of this reduction remained unknown. As the reduction of survivin expression by brexpiprazole was partially rescued by the administration of MG132, a proteasome inhibitor, the involvement of proteasome degradation in the regulation of survivin levels is suspected. The expression of survivin was reported to be regulated by the ubiquitin-proteasome pathway, and the ubiquitination status of survivin is regulated by E3 ligases: FBXL7 [34, 35] and cullin 9 [36, 37], and by a deubiquitinase, USP9X [38–40]. In addition, the correct folding of survivin by Hsp90 prevents proteasomal degradation [41]. To elucidate the detailed mechanism of regulation of survivin by antipsychotic drugs, further studies focusing on the mechanisms of survivin degradation, including ubiquitination and folding by Hsp90, are needed.

Recently, monoamines, such as serotonin and dopamine, are attracting attention as targets for cancer therapy [42–45]. Alteration of monoamine activity in cancer cells is suspected to be involved in the mechanism of the anti-tumor activity of olanzapine, aripiprazole, and brexpiprazole ([24, 25] and this study) because all three drugs target monoamine receptors. However, their anti-cancer effects were not rescued or mimicked by treatment with monoamines or antagonists of monoamine receptors, respectively (data not shown). To elucidate the mechanism, further studies are needed.

In this study, we revealed that brexpiprazole can inhibit cancer cell growth *in vitro* and *in vivo*, induce cytotoxic cell death in cancer cells, and reduce CSC properties and the expression of survivin, which is a known chemoresistant factor. In conclusion, brexpiprazole is a promising anti-tumor drug with an improved side effect profile, as compared with previously reported antipsychotic drugs, and is a potential candidate drug for patients with cancer.

## MATERIALS AND METHODS

### Antibodies and reagents

Anti-Sox2 (#3579), anti-Nanog (#4903), anti-Bmi1 (#2830), and anti-Survivin (#2808) antibodies were purchased from Cell Signaling Technology, Inc. (Beverly, MA, USA). Anti-β-actin (A1978) antibody was from Sigma (St. Louis, MO, USA). Anti-CD133 antibody (W6B3C1) was from Miltenyi Biotec (Germany). Gemcitabine and 5-fluorouracil (5-FU) were from Sigma, and were dissolved in DMSO to prepare 1 mM and 200 mM stock solutions, respectively. Brexpiprazole was from Cayman Chemical (Ann Arbor, MI, USA) and dissolved in DMSO to prepare a 10 mM stock solution.

### Cell culture

The human non-small cell lung cancer (NSCLC) cell line A549 was obtained from the Riken BioResource Center (Tsukuba, Japan). The human pancreatic cell line PANC-1 was from the Cell Resource Center for Biomedical Research, Institute of Development, Aging and Cancer, Tohoku University (Sendai, Japan). In addition, another human pancreas cancer cell line, PSN-1, was a kind gift from Dr. Teruhiko Yoshida at the National Cancer Center Research Institute [46]. These cell lines were cultured in DMEM/F12 medium supplemented with 10% fetal bovine serum (FBS), 100 units/mL of penicillin, and 100 μg/mL of streptomycin. The establishment of A549 CSLC, PANC-1 CSLC, and PSN-1 CSLC cell lines was previously reported [47–52]. GS-Y03 is a CSC line derived from glioma [53]. The authenticity of A549 CSLC, PANC-1 CSLC, and PSN-1 CSLC cell lines was verified by genotyping of short tandem repeat (STR) loci (Bio-Synthesis, Inc., Lewisville, TX, USA) and comparing with the ATCC STR database for Human Cell Lines. These CSLC cells were cultured as previously described [48–53]. Briefly, these cells were cultured on collagen I-coated dishes (IWAKI, Tokyo, Japan) in the stem cell culture medium (DMEM/F12 medium with 1% B27 supplement [Gibco-BRL, Carlsbad, CA, USA], 20 ng/mL of EGF and FGF2 [Peprotech, Inc., Rocky Hill, NJ, USA], D-(+)-glucose [final concentration, 26.2 mM], L-glutamine [final concentration, 4.5 mM], 100 units/mL of penicillin, and 100 μg/mL of streptomycin). The stem cell culture medium was changed every 3 days, and EGF and FGF2 were added to the culture medium every day. IMR90, normal human fetal lung fibroblasts [24, 25, 54, 55], were purchased from American Type Culture Collection, and cultured in DMEM/F12 supplemented with 10% FBS, 100 units/mL of penicillin, and 100 μg/mL of streptomycin. All experiments with IMR90 were carried out within a low passage number (less than nine). Rat cortical stem cells were obtained from R&D systems (Minneapolis, MN, USA). They were cultured on a plate coated with Geltrex (Thermo Fisher Scientific, Waltham, MA, USA) under the stem cell culture conditions (11, 12). All rat cortical stem cell experiments were performed using a low passage number (less than nine).

### Sphere formation assay

After dissociating into single cells, CSCs were serially diluted in the stem cell culture medium and seeded on non-coated 96-well plates to be a single cell in each well. Wells containing a single cell were marked on the next day, and 1 week after seeding, the percentage of marked wells with a sphere relative to the total number of marked wells was calculated [24, 25, 48].

### Cell viability assays

Viable and dead cells were identified by their ability and inability to exclude vital dyes, respectively [24, 25, 52, 53]. Briefly, cells were stained with 0.2% trypan blue, and the numbers of viable and dead cells were counted using a hemocytometer. Dead cells (%) was defined as 100× ‘the number of dead cells’ / (‘the number of viable cells’ + ‘the number of dead cells’).

### Immunoblot analysis

Cells were washed with PBS and lysed in RIPA buffer (10 mM Tris-HCl [pH 7.4], 0.1% SDS, 0.1% sodium deoxycholate, 1% NP-40, 150 mM NaCl, 1 mM EDTA, 1.5 mM Na_3_VO_4_, 10 mM NaF, 10 mM sodium pyrophosphate, 10 mM sodium β-glycerophosphate, and 1% protease inhibitor cocktail set III Sigma]. After centrifugation for 10 minutes at 14,000 × g at 4°C, the supernatants were harvested as the cell lysates, and the protein concentration of the cell lysates was measured using a BCA protein assay kit (Pierce Biotechnology, Inc., Rockford, IL, USA). Cell lysates containing equal amounts of protein were separated by SDS-PAGE and transferred to polyvinylidene difluoride membranes. The membranes were probed with primary antibodies and then with an appropriate HRP-conjugated secondary antibody according to the manufacturer’s protocol. Immunoreactive bands were visualized using Immobilon Western Chemiluminescent HRP Substrate (Merck Millipore, Darmstadt, Germany).

### Flow cytometric analysis

Flow cytometry was conducted as previously described [48]. Briefly, dissociated cells were washed with ice-cold PBS, fixed with 4% (w/v) paraformaldehyde for 10 min at room temperature (RT), and washed again with ice-cold PBS. The cells were blocked in FCM buffer (0.5% [w/v] bovine serum albumin, 0.1% [w/v] NaN_3_ in PBS) for 1 hour, followed by three PBS rinses and further incubation with anti-CD133 antibody in the FCM buffer overnight at 4°C, and then with the Alexa Fluor^®^ 488 goat anti-mouse IgG (Thermo Fisher Scientific) for another 1 hour at RT. Single cells were gated using forward scatter in the isotype control samples. The isotype control samples were used to establish a gate in the fluorescein isothiocyanate channel. Cells exhibiting signals for CD133 above the gate established by the isotype control were considered CD133-positive cells. Flow cytometry experiments were performed using the FACSCanto^TM^ II Flow Cytometer (BD Biosciences, Franklin Lakes, NJ, USA). Data were analyzed by FlowJo^®^ software, version 7.6.5 (Treestar Inc., Ashland, OR, USA).

### RNA extraction and reverse transcription-PCR

Total cancer cell RNA was isolated using Trizol (Thermo Fisher Scientific) according to the manufacturer’s protocol. RNA was reverse transcribed into cDNA using the PrimeScript 1st strand cDNA Synthesis Kit (Takara Bio, Kusatsu, Japan). Synthesized cDNA samples were quantified by PCR using Quick Taq HS DyeMix (Toyobo, Osaka, Japan). The primer sequences were; actin (5′-CTTAGTTGCGTTACACCCTTTCT-3′ [forward] and 5′-CTGCTGTCACCTTCACCGTTCC-3′[reverse]), and survivin (5′-TTGTCGACACCATGGGTGCCCCG-3′ [forward] and 5′-TTTTGCGGCCGCTCAATCCATGG-3′ [reverse]).

### Mouse study

Mouse xenograft studies were carried out as previously described [56, 57]. For subcutaneous implantation, PANC-1 CSLC cells (1 × 10^5^ implanted cells) suspended in 200 μL of PBS were implanted subcutaneously into the bilateral flank regions of 5- to 8-week-old male BALB/cAJcl-*nu*/*nu* mice (CLEA Japan, Inc., Tokyo, Japan) after being anesthetized by intraperitoneal injection of medetomidine, midazolam, and butorphanol (0.3 mg, 4 mg, and 5 mg per kg body weight, respectively). After implantation, the general health status and the presence of subcutaneous tumors were monitored. The tumor volume was calculated by measuring tumor diameters (measurement of 2 perpendicular axes of tumors) using a caliper and calculated as 1/2 × (larger diameter) × (smaller diameter)^2^. For systemic administration of brexpiprazole, a stock solution of brexpiprazole (4 mg/mL in DMSO) was diluted in DMSO to prepare 100 μL solutions for each injection. The brexpiprazole was administered intraperitoneally to mice at 0.1 mg/kg body weight three times a week. Drug treatment was initiated after confirmation of subcutaneous tumor formation, and tumor-bearing mice were randomized into two groups before the initiation of drug treatment. All animal experiments were performed following a protocol approved by the Animal Research Committee of Yamagata University.

### Immunohistochemisty

The excised subcutaneous tumors were fixed in 4% paraformaldehyde for 24 hours at 4°C, and paraffin sections were prepared. Immunohistochemical staining was performed using Histofine Simple Stain MAX PO (Nichirei biosciences, Tokyo, Japan) and ImmPACT DAB peroxidase substrate (Vector Laboratories, Burlingame, CA, USA) on deparaffinised sections treated with 10 mM Tris-HCl (pH 10.0). The anti-Sox2 (Cell Signaling Technology Inc.) and anti-Survivin antibodies (Cell Signaling Technology Inc.) were used as primary antibodies. Representative images were taken using a BZ-X800 microscope (KEYENCE, Osaka, Japan).

### Statistical analysis

The results are expressed as the means and standard deviation (SD). The differences were compared using the two-tailed Student’s *t*- test. *P*-values < 0.05 were considered significant and indicated with asterisks.
